# Supplementary figures and images for: Integrating remote monitoring into heart failure patients’ care regimen: A pilot study
Source: PLoS One. 2020 Nov 19;15(11):e0242210. doi: 10.1371/journal.pone.0242210 (PMC7676713; doi:10.1371/journal.pone.0242210)

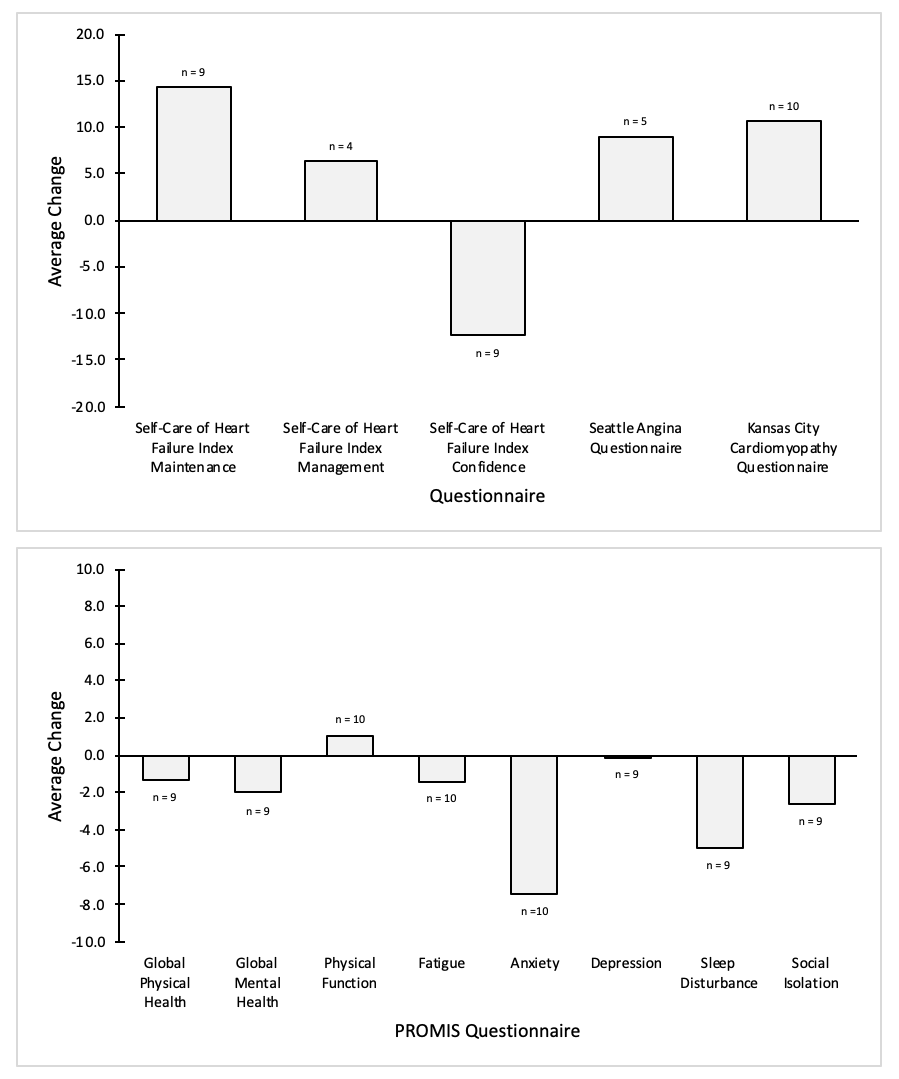

Supplement: S1 Fig — For non-PROMIS questionnaires, a positive change indicates improvement in health status. A positive change also signifies improvement in health status for the following PROMIS questionnaires: Global Physical Health, Global Mental Health, and Physical Function. Conversely, a negative change is indicative of improvement in health status for the following PROMIS questionnaires: Fatigue, Anxiety, Depression, Sleep Disturbance, and Social Isolation. (TIF) [file pone.0242210.s002.tif]

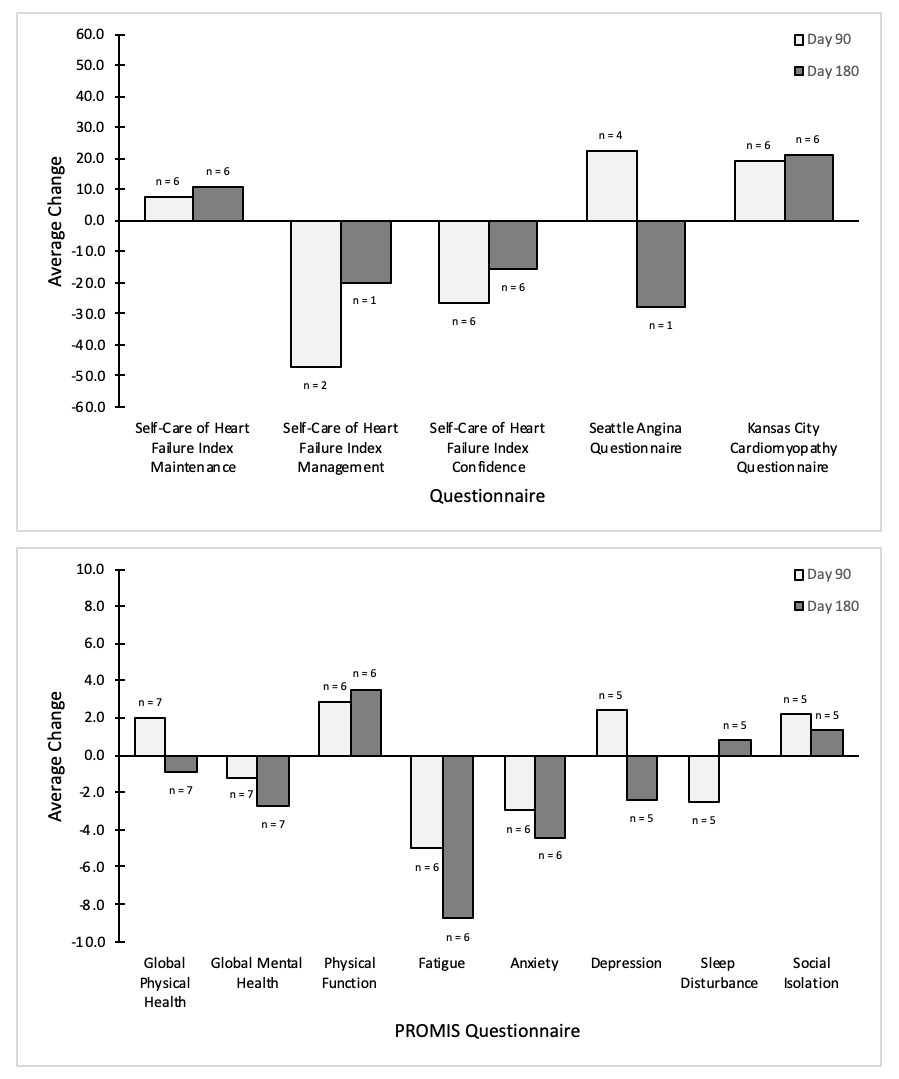

Supplement: S2 Fig — For non-PROMIS questionnaires, a positive change indicates improvement in health status. A positive change also signifies improvement in health status for the following PROMIS questionnaires: Global Physical Health, Global Mental Health, and Physical Function. Conversely, a negative change is indicative of improvement in health status for the following PROMIS questionnaires: Fatigue, Anxiety, Depression, Sleep Disturbance, and Social Isolation. (TIF) [file pone.0242210.s003.tif]
